# Research on the influence of different sampling resolution and spatial resolution in sampling strategy on landslide susceptibility mapping results

**DOI:** 10.1038/s41598-024-52145-w

**Published:** 2024-01-18

**Authors:** Xianyu Yu, Huihui Chen

**Affiliations:** 1 School of Civil Engineering, Architecture and Environment, Hubei University of Technology, Wuhan, 430068 China; 2 Innovation Demonstration Base of Ecological Environment Geotechnical and Ecological Restoration of Rivers and Lakes, Hubei University of Technology, Wuhan, 430068 China

**Keywords:** Environmental sciences, Natural hazards

## Abstract

Landslides, recognized as a significant global natural disaster, necessitate an exploration of the impact of various resolution types in sampling strategies on Landslide Susceptibility Mapping (LSM) results. This study focuses on the segment from Zigui to Badong within the Three Gorges Reservoir Area, utilizing two resolution types: sampling resolution and spatial resolution, The Support Vector Machine (SVM) is employed to obtain LSM results, which are then analyzed using Receiver Operating Characteristic (ROC) curve, specific category accuracy and statistical methods. Artificial Neural Network (ANN) and Convolutional Neural Network (CNN) were used to verify the reliability of the results. Additionally, five common machine learning models, including Logistic Regression (LR), are used to conduct experiments on four sampling resolutions (10 m,30 m,50 m and 70 m) to further investigate the effect of sampling resolution on LSM results. These are evaluated using a comprehensive quantitative method. The results reveal that increasing spatial resolution improves the prediction accuracy, while increasing sampling resolution produces a contrary effect. Furthermore, the impact of spatial resolution on LSM results is more pronounced than that of sampling resolution. Finally, Fanjiaping landslide and Huangtupo landslide are selected as references for comparative analysis, with the results aligning with engineering reality.

## Introduction

A landslide is the process in which a hillside body composed of rock, soil, or debris accumulations is subjected to the combined effects of groundwater activity, seismic activity, or artificial slope-cutting, and under the action of gravity, an overall downward slide occurs along a certain soft and weak surface^1^. As one of the most frequent types of geological hazards, landslides result in significant losses to human life, the economy, and other areas annually. The occurrence of landslides is influenced by numerous factors, including landform, geology, soil composition, weather conditions, and land use^2^. Due to their wide distribution, high frequent, destructive nature, and rapid development, landslides present unique characteristics. They can also trigger secondary disasters such as river blockages, leading to floods and mudslides, further intensifying the damage^3^. Consequently, landslides, as significant geological events, have profound implications for human survival and development^4^.

Advancement in Geographic Information System (GIS) and earth observation technology have made Remote Sensing (RS) and GIS essential in modeling natural disaster susceptibility^5^. Landslide Susceptibility Mapping (LSM) is a commonly employed approach for predicting the spatial distribution and probability of landslides. LSM outcomes are crucial for reducing landslide disaster risks and for efficient land resource allocation^6^. In LSM modeling, the sampling strategy significantly affects the results. Several studies have validated this impact. Liu et al. proposed a frequency-ratio based LSM sampling strategy, showing superior performance over conventional methods^7^. Tekin et al. assessed the effects of two landslide sampling techniques on LSM, finding that selecting landslide-affected pixels from the entire landslide polygon yields higher prediction accuracy than selecting a similar proportion of pixels from any part of the landslide body^8^. Dagdelenler et al. re-evaluated seed cell sampling strategies, presenting the effects of two different sampling strategies (landslide zones and seed cells) on LSM and comparing the susceptibility maps derived from these strategies^9^. Hussin et al. summarized four common landslide sample extraction strategies: (1) using the center-of-mass method for individual pixel sampling; (2) extracting all pixels within the entire landslide body; (3) selecting pixel points in and around the landslide crown line using the main scarp upper edge method; (4) the seed-cell approach^10^. However, these strategies focus solely on various landslide sampling methods, and only a small amount of literature has considered the consistency of non-landslide and landslide sampling methods^11,12^.

Different resolutions of samples serve as a global sampling strategy, applying the same method for both landslides and non-landslides. Sample resolution includes spatial and sampling resolution. Regarding spatial resolution, the prediction accuracy of landslide susceptibility heavily relies on the quality of input data, primarily derived from Digital Elevation Models (DEM)^13^. Therefore, selecting the appropriate DEM spatial resolution is a crucial step in LSM research^14^. Schlögel et al. conducted LSM in the Ubaye Valley of the southern French Alps using three different resolutions (5 m, 10 m, and 25 m) for slope units, with their findings favoring a 10 m resolution^15^. Meena et al. evaluated LSM in the Kulu Valley of the Himalayan Mountains using three different spatial resolutions (12.5 m, 30 m, and 90 m), highlighting the highest accuracy at a 30 m resolution^16^. Chen et al. assessed the impact of seven spatial resolutions ranging from 30 to 90 m on LSM prediction and identified the highest accuracy at a 70 m resolution. Their study concluded that a finer resolution did not necessarily yield superior accuracy in LSM prediction^14^. However, studies regarding the potential impact of sampling resolution on LSM have not been reported in the literature. Based on the available literature, it can be concluded that the effect of spatial resolution on LSM has no obvious regularity in different study areas and spatial resolution. Additionally, there is a notable gap in research concerning the influence of sampling resolution on LSM, so there is no unified standard for the potential effect of different resolutions on LSM.

This article takes focuses on Zigui to Badong section of in the Three Gorges Reservoir Area to investigates the influence of different sampling resolutions (10 m, 16 m, and 30 m) and spatial resolutions (10 m, 16 m, and 30 m) on LSM outcomes. Nine LSM factors, including elevation, slope, aspect, curvature, lithology, distance to faults, Topographic Wetness Index (TWI), Normalized Difference Vegetation Index (NDVI), and multi-year average rainfall. The experiment on sampling resolution selected LSM factors using a 16 m × 16 m and 30 m × 30 m window based on a fixed LSM factor resolution of 10 m. To correspond with the selected sampling resolution, the experiment on spatial resolution used corresponding DEM (10 m, 16 m, and 30 m) and remote sensing images [Sentinel-2 (10 m), GF-1 (16 m), and Landsat-8 (30 m)] to obtain the LSM factors. For the experiment, all the grid points in the study area were used as the whole sample, the grid points in 70% of the landslide surface and an equivalent number of non-landslide grid points were randomly selected to construct the training set, while the grid points in the remaining 30% of the landslide surface serves as a validation set. The support vector machine (SVM) is used to derive LSM results at different sampling and spatial resolutions, evaluated using the Receiver Operating Characteristic (ROC) curve, specific category accuracy and statistical methods. To ensure the reliability of the experimental findings, both artificial neural network (ANN) and convolutional neural network (CNN) are employed, and a comprehensive quantitative scoring method evaluates the LSM results of the three models. In order to further explore the impact of sampling resolution on LSM results, five common machine learning models, including logistic regression (LR), were used to conduct experiments on different sampling resolutions (10 m, 30 m, 50 m, and 70 m), which consistently support the conclusions the previous experiment. Fanjiaping and Huangtupo landslides are selected for comparative analysis and verification, with results aligning with engineering reality. This comprehensive investigation of optimal sampling and spatial resolutions aims to enhance the scientific precision and accuracy of LSM, offering significant theoretical and practical value.

**Figure 1:**
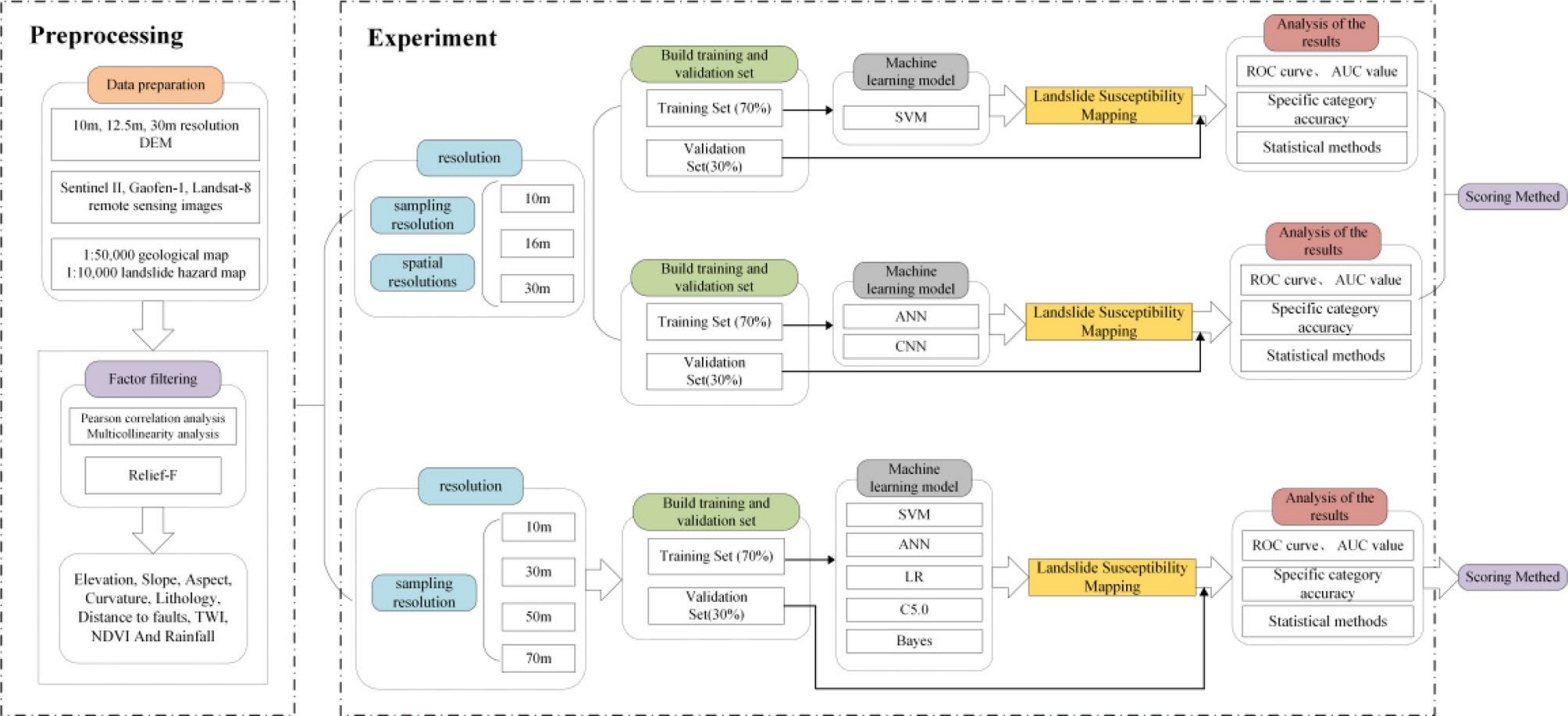
Experimental flow chart.

The flow chart of this article is shown in Fig. 1.

## Study area and data sources

### Study area

**Figure 2:**
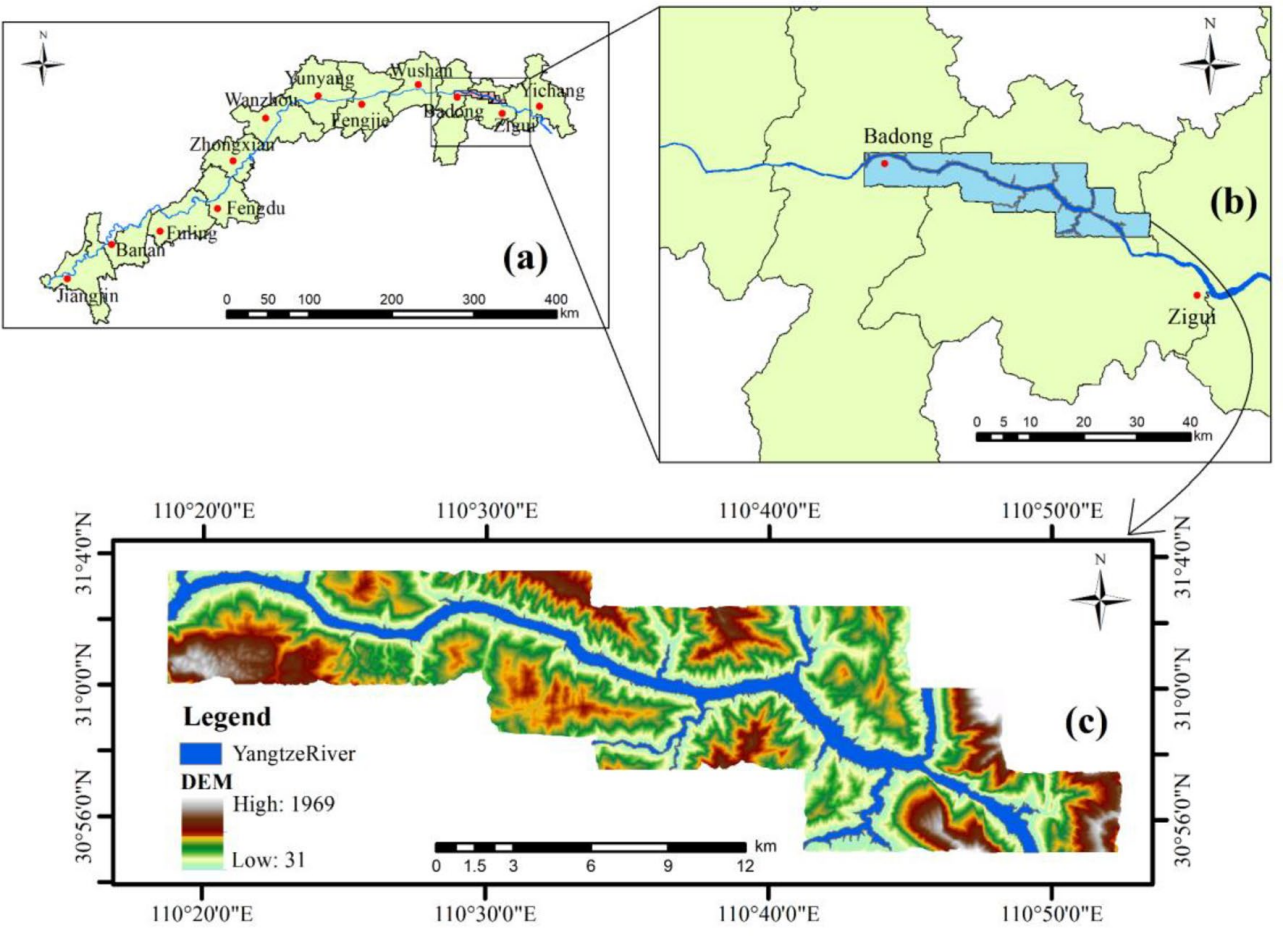
(**a**) Schematic map of the Three Gorges reservoir area. (**b**) Schematic map of the study area crossing boundaries. (**c**) Schematic map of the elevation of the study area.

The study area, situated in the Zigui to Badong section, represents the first area of the Three Gorges Reservoir of the Yangtze River, spans 55 km in length and covers an area of 388 square kilometers, situated at 100°18′–110°52′ east longitude and 30°01′–30°56′ north latitude. A schematic diagram of the study area is provided in Fig. 2. Located within the mid-latitude subtropical monsoon climate zone, the area’s climatic elements are influenced by local topography and elevation variations. exhibiting notable spatial and temporal distribution variations significant microclimate characteristics^17^. Geological hazards primarily include landslides, collapses, and bank collapses, with landslides being particularly frequent, causing substantial human casualties and economic losses. Typical landslides in the area include Fanjiaping, Zhaoshuling, Xintan, Baishuihe, among others^18^.

### Data

#### Raw data

The main data used in this article and their applications are detailed in Table 1. The 1:50,000 scale geological map^19^ and the 1:10,000 scale landslide hazard map^20^ can meet the precision requirements of 10 m resolution, and can match the highest precision DEM data and remote sensing image data in this article. The landslide hazard database includes information about the occurrence time, type, and impact of some landslides in the study area. The rainfall data, derived from interpolation at each rainfall monitoring station, which only has time resolution but not spatial resolution, so the annual average rainfall for many years is used to eliminate the influence of time factors. It should be noted that, for consistency in resolution and facilitating a comprehensive analysis of the impact of spatial resolution and sampling resolution on LSM results, the 16 m DEM data utilized in this study were obtained by resampling the global 12.5 m DEM data provided by the ALOS satellite.

**Table 1:** Data list of the study area.

Name	Spatial resolution/scale	Use
DEM data	10 m, 12.5 m, 30 m	Extract elevation, slope, aspect, TWI and other factors
Sentinel-2 GF-1 Landsat-8	Multispectral 10 m Multispectral 16 m Multispectral 30 m	Extract NDVI
Basic geological map	1:50,000	Extract lithology
Landslide hazard map	1:10,000	Extract landslide information
Landslide hazard database	–	Extract partial landslide time, type, damage, etc
Rainfall data	–	Get average annual rainfall information

#### Factor resolution processing

The factorial resolutions are treated as follows (with ① and ④ being the same experimental data):

- ① 10 m sampling resolution (SA-10): the basic LSM factors resolution is 10 m, achieved by employing a 10 m DEM and Sentinel-2 (10 m) remote sensing images to obtain the LSM factors at the corresponding sampling resolution.
- ② 16 m sampling resolution (SA-16): on the basis of the same LSM factors resolution (10 m), the LSM factors are selected using a 16 m × 16 m window.
- ③ 30 m sampling resolution (SA-30): on the basis of the same LSM factors resolution (10 m), the LSM factors are selected using a 30 m × 30 m window.
- ④ 10 m spatial resolution (SP-10): the basic LSM factors resolution is 10 m, achieved by employing a 10 m DEM and Sentinel-2 (10 m) remote sensing images to obtain the LSM factors at the corresponding spatial resolution.
- ⑤ 16 m spatial resolution (SP-16): the LSM factors are resolved at a spatial resolution of 16 m, obtained through resampling a 12.5 m DEM to a 16 m DEM and GF-1 (16 m) remote sensing images to obtain the LSM factors at the corresponding spatial resolution.
- ⑥ 30 m spatial resolution (SP-30): the LSM factors are resolved at a spatial resolution of 30 m, achieved by employing a 30 m DEM and Landsat-8 (30 m) remote sensing images to obtain the LSM factors at the corresponding spatial resolution.

#### Factor data

**Figure 3:**
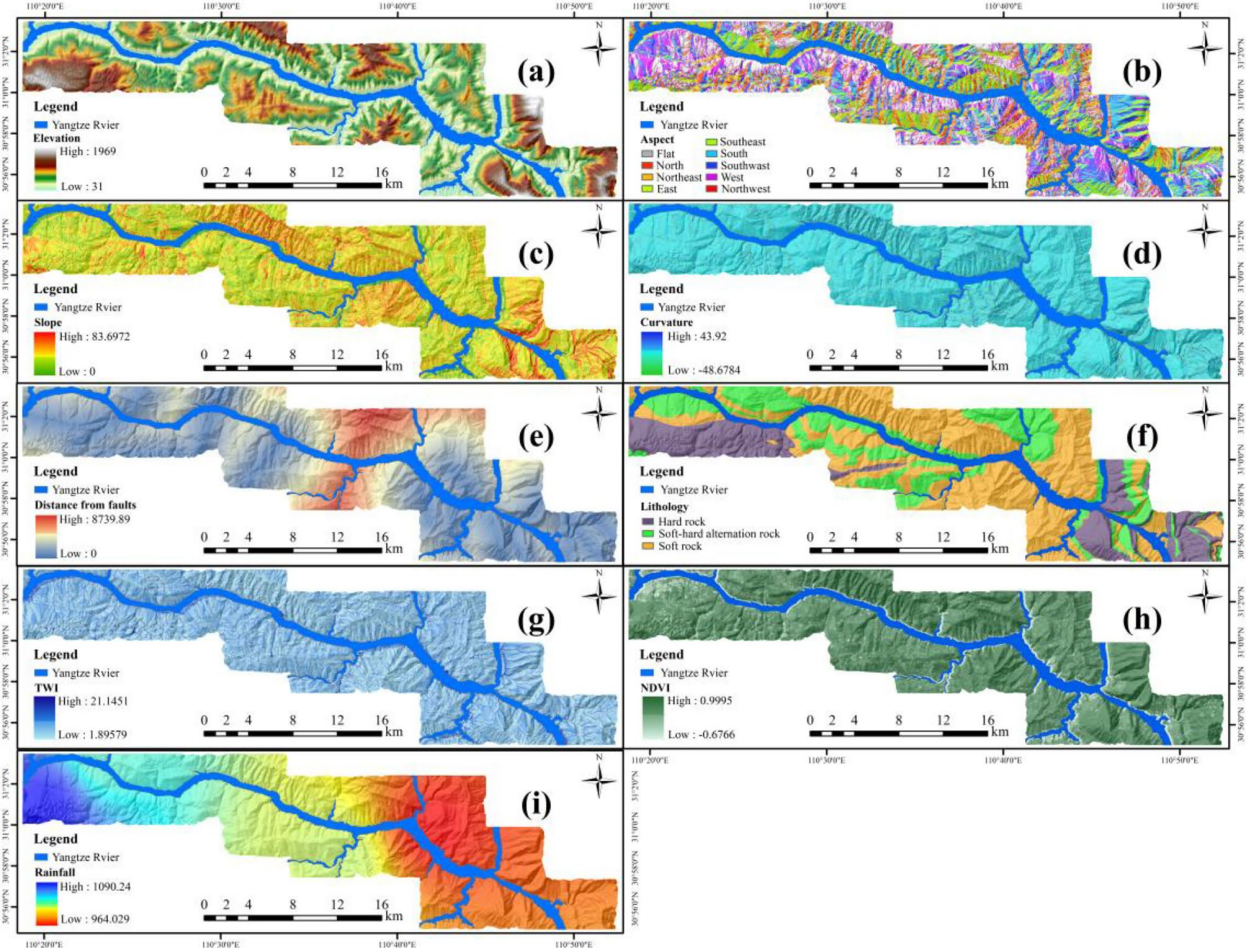
LSM factors in the study area: (**a**) elevation, (**b**) aspect, (**c**) slope, (**d**) curvature, (**e**) distance from faults, (**f**) lithology, (**g**) TWI, (**h**) NDVI, (**i**) rainfall.

Based on the literature review, nine commonly used base factors are selected for this study: elevation, slope, aspect, curvature, lithology, distance to faults, TWI, NDVI, and multi-year average rainfall^6,21,22^. To avoid correlation and multicollinearity among these factors, diagnostic methods like Pearson Correlation Coefficient^23^, Variance Inflation Factor and Tolerance^24^, and Relief-F algorithms^25^ are employed. The final diagnosed landslide evaluation factors are shown in Fig. 3, with related information presented in Table 2.

**Table 2:** LSM factor-related information.

Category	Factors	Unit	Value range
Topography	Elevation	m	31–1969
	Aspect	–	(1) Flat; (2) north; (3) northeast; (4) east; (5) southeast; (6) south; (7) southwest; (8) west; (9) northwest
	Slope	m	0–83.6972
	Curvature	–	−48.6784 to 43.92
Geological	Distance from faults	m	0–8739.89
	Lithology	–	(1) Hard rock; (2) soft–hard alternating rock; (3) soft rock
Hydrography	TWI	–	1.89579–21.1451
Human engineering activities	NDVI	–	−0.6766 to 0.9995
Atmospheric rainfall	Rainfall	mm/year	964.029–1090.24

### Software

The software used in this article includes ArcGIS 10.8, ENVI 5.3, IBM SPSS Statistics 26, IBM SPSS Modeler 18, and PyTorch 1.7.1. The sources and uses are shown in Table 3.

**Table 3:** Sources and uses of software.

Name	Source	Use
ArcGIS 10.8	https://www.esri.com/	Landslide susceptibility mapping
ENVI 5.3	https://envi.geoscene.cn/	Remote sensing image processing
IBM SPSS Statistics 26	https://www.ibm.com/	Data analysis
IBM SPSS Modeler 18	https://www.ibm.com/	SVM and ANN modeling
PyTorch 1.7.1	https://pytorch.org/	CNN modeling

## Experimental models and methods

### Models

#### SVM model

SVM, initially proposed by Vapnik^26^, is a supervised learning method utilized for classification, regression, and anomaly detection^27^. Known for its high prediction accuracy and performance, SVM is considered a classic nonlinear prediction model for evaluation^28^. Assuming a linearly separable training vector *x*_*i*_ (*i* = 1, 2, …, *n*) belonging to two different classes *y*_*i*_ =  ± 1, SVM can find an n-dimensional hyperplane in the data space using a kernel function, so that the margin ${{\parallel w\parallel } \mathord{\left/ {\vphantom {{\parallel w\parallel } 2}} \right. \kern-0pt} 2}$ between the classification boundary and the nearest data point is the largest, thereby clearly distinguishing between landslide and non-landslide categories^29^. This hyperplane is represented by formulas (1) and (2).

1 $$L = \frac{1}{2}\left\| w \right\|^{2}$$

2 $$y_{i} ((w \times x_{i} ) + b) \ge 1$$

where $\parallel w\parallel$ is the normal vector norm of the hyperplane, *b* is a scalar, *x*_*i*_ is a point on the hyperplane, and *w* is a vector perpendicular to the hyperplane.

Commonly employed kernel functions include linear, polynomial, Radial Basis Function (RBF), and Sigmoid. Among these, RBF demonstrates superior performance with fewer parameters and greater flexibility^30^. Therefore, this study adopts an RBF kernel-based SVM approach for LSM.

**Figure 4:**
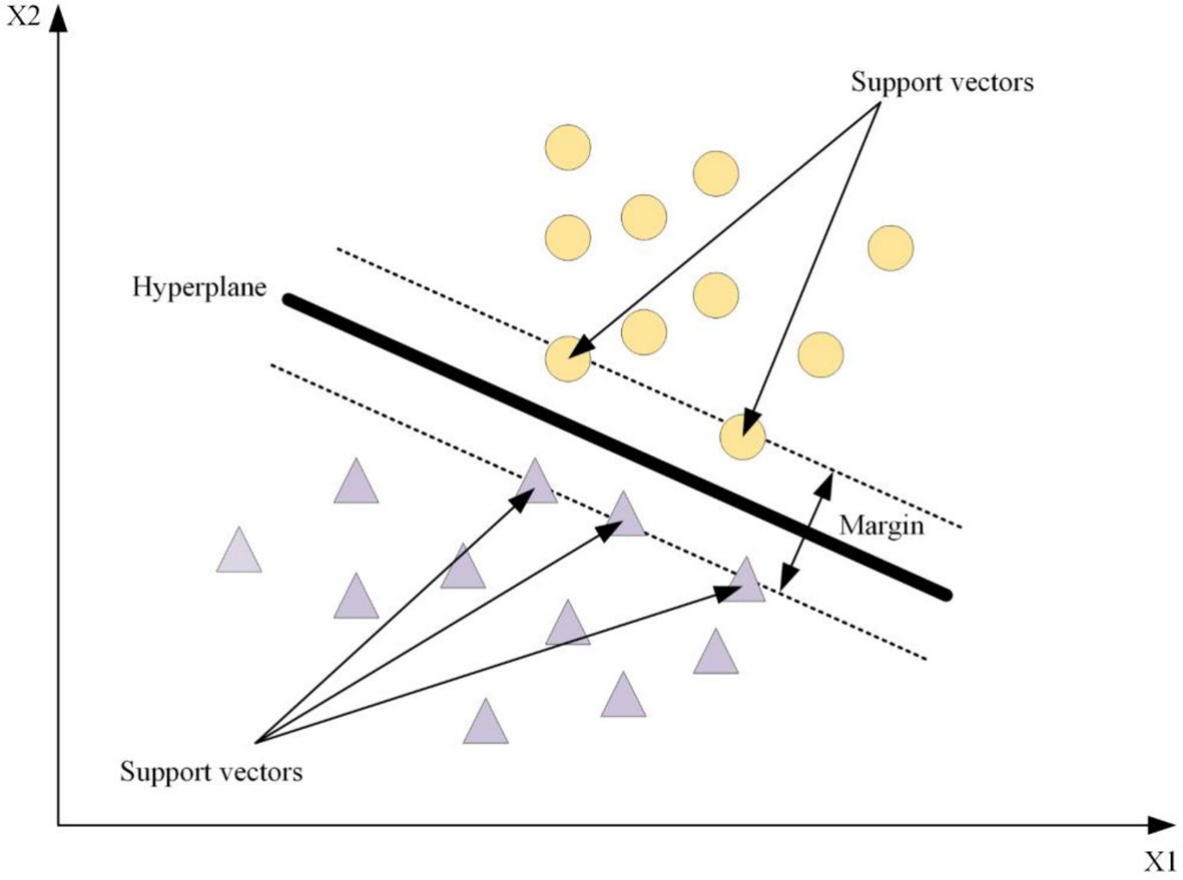
Schematic map of the SVM model.

The schematic map of the SVM model is shown in Fig. 4.

#### ANN model

**Figure 5:**
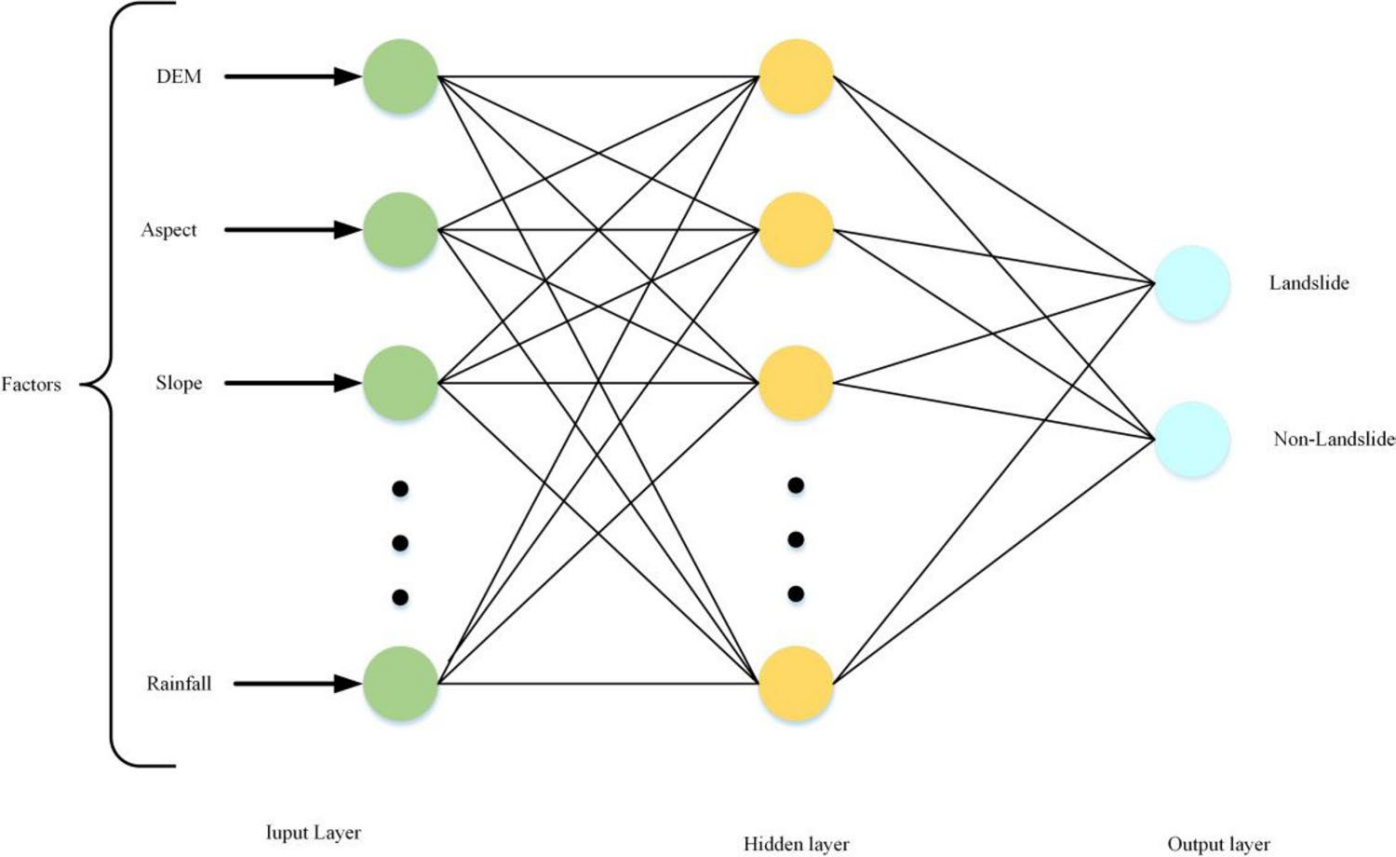
Schematic map of the ANN model.

ANN is a nonlinear computational model that mimics the human nervous system for information acquisition, processing, representation, and calculation^31^. The ANN model offers several advantages: (a) strong generalization ability, (b) robust self-learning capability and adaptability, (c) excellent nonlinear mapping capability, (d) high fault tolerance and good fitting performance^28^. Typically, the ANN model consists of three interconnected layer types: input layer, hidden layer, and output layer^29^, as shown in Fig. 5. In this article, the input layer represents the LSM factors, the hidden layer encompasses the neurons utilized, and the output layer signifies the predicted likehood of landslide occurrence along with the calculation of its probability value.

#### CNN model

**Figure 6:**
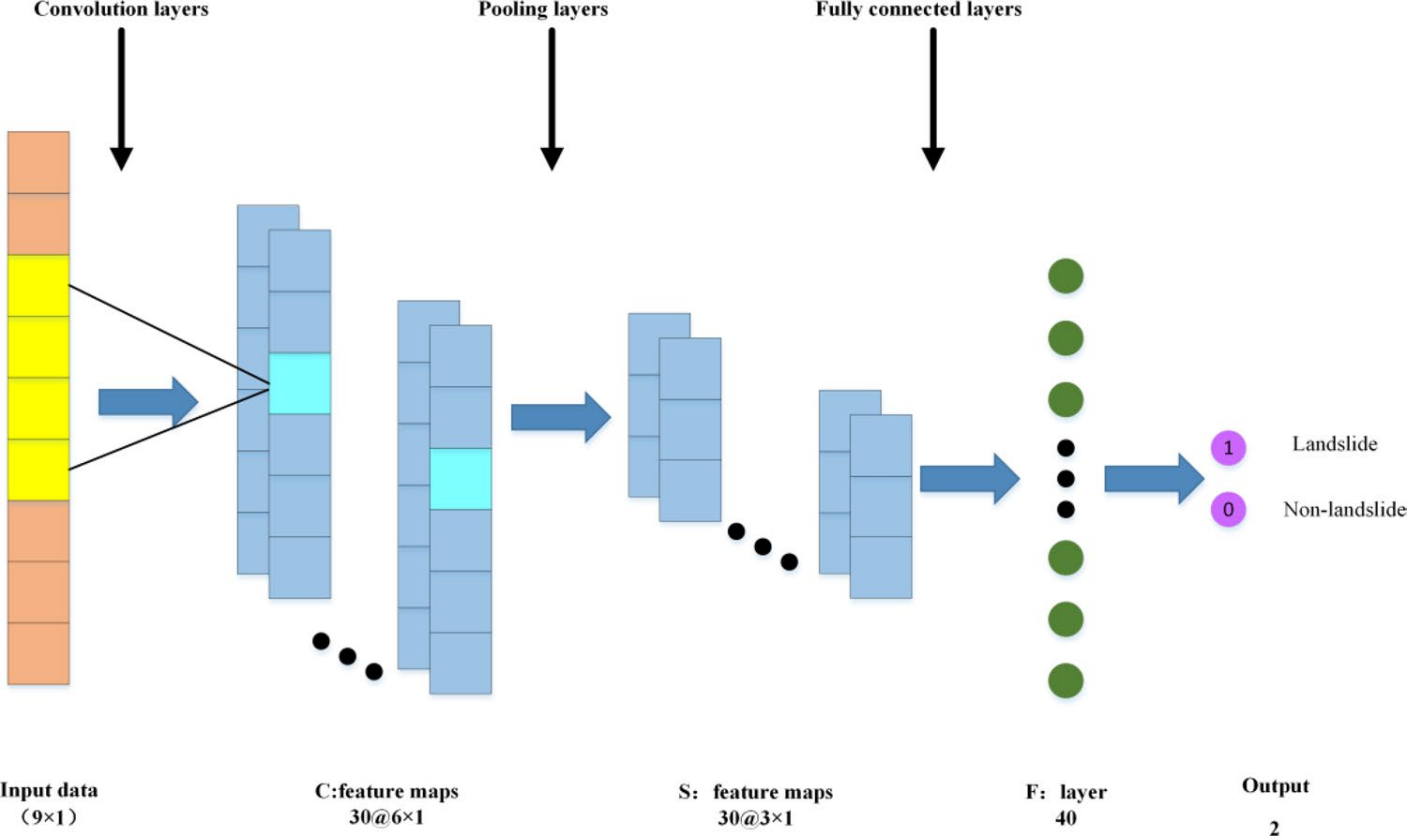
Schematic map of the CNN model.

The deep learning algorithm CNN, introduced by LeCun et al.^32^, is an enhanced approach based on ANN, where artificial neurons respond to surrounding elements to extract information^33^. A typical CNN model includes five essential components: the input layer, convolutional layer, pooling layer, fully connected layer, and output layer^2^, as shown in Fig. 6. The convolutional layer, central to CNN, consists of multiple convolutional kernels that linearly map the input data to extract finer feature information. The adoption of a shared weight strategy in the convolutional layer allows the entire network to be trained with fewer parameters compared to a fully connected network^34^. The pooling layer crucial in CNN, performs downsampling operations through various nonlinear functions to reduce feature size, retain essential details, and mitigate overfitting with different data^35^. The fully connected layer acts as a “classifier” within the convolutional neural network, with its input comprising high-dimensional features extracted after the operations of the convolutional and pooling layers^36^. The parameters of CNN used in this article are shown in Table 4.

**Table 4:** CNN parameter values.

Various parameters	Parameter value	Various parameters	Parameter value
Convolution kernel size	1 × 3	Iterations	30
Max pooling kernel	1 × 2	Batch data size	2000
Activation function	ReLu	Learning rate	0.0001
Error function	Cross entropy error	Optimizer	Adam

### Evaluation methods

#### ROC curve

**Figure 7:**
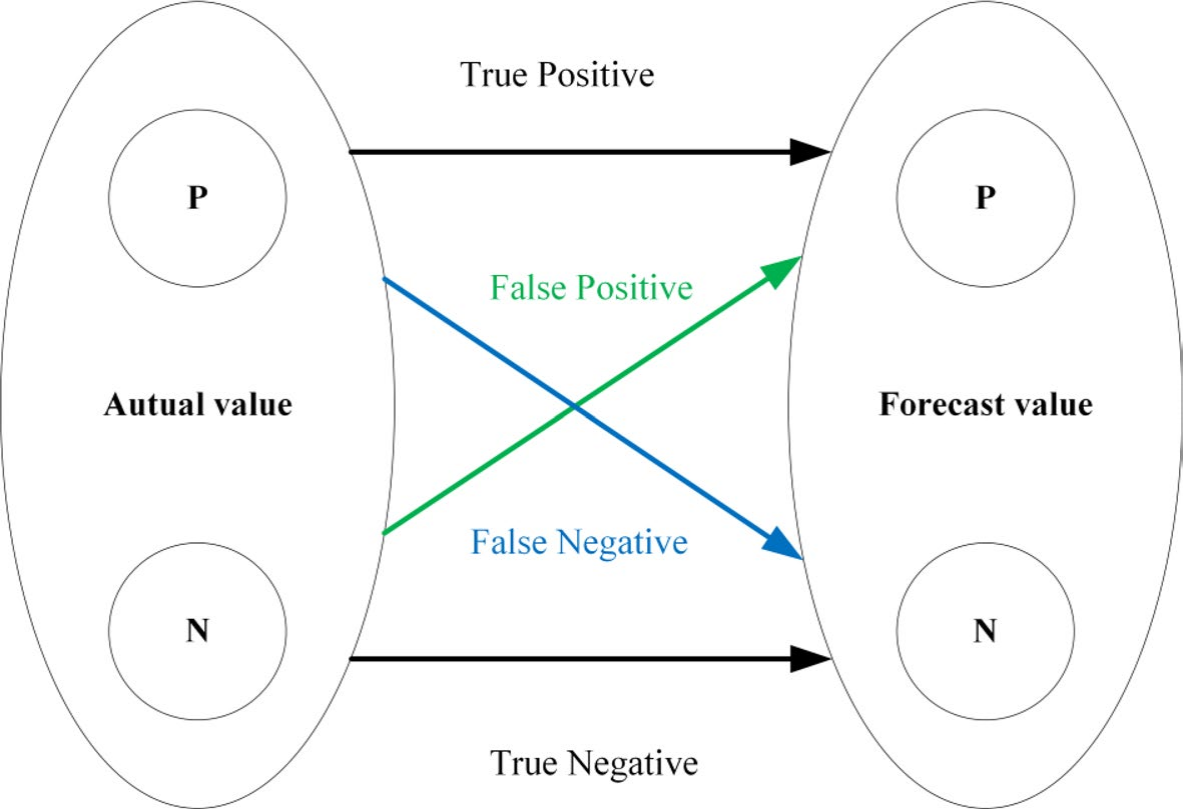
Schematic map of the dichotomous model.

ROC curve is widely employed for analyzing LSM results^37–39^. The ROC curve focuses on binary classification model, with four possible outcomes in prediction results: (1) True Positive (TP), (2) False Positive (FP), (3) True Negative (TN), (4) False Negative (FN). These results can be represented by a confusion matrix, as shown in Fig. 7.

Where P is a positive example and N is a negative example^40^.

The ROC curve begins at the point (0, 0) and ends at (1, 1), plotting the true negative rate (TNR) represented on the horizontal axis and the true positive rate (TPR) depicted on the vertical axis^41^. This article evaluates LSM results at different sampling and spatial resolutions through ROC curves and AUC values.

#### Specific category precision analysis

The conventional approach for quantitative analysis in LSM relies on Landslide Susceptibility Zoning (LSZ) results, calculated by the ratio of landslide area in highly susceptible zones to total landslide area. The specific category precision analysis method, however, considers the number of calculation units within classified zones, thus addressing the issue of the model producing favorable results when a large portion of the LSZ falls within the extremely high-risk category. This method provides a more suitable approach for LSM results analysis^42^. As shown in formula (3).

3 $$P_{i} = \frac{{A_{i} }}{{B_{i} }} \times 100\%$$

where *i* = 1, 2, …, *n*, *n* is the number of landslide-prone zoning categories, *A*_*i*_ is the number of grid cells occupied by landslides in the *i*-th landslide-prone zoning category, *B*_*i*_ is the number of landslides in the *i*-th landslide-prone area category, and *P*_*i*_ is the specific category precision of the *i*-th landslide-prone area category.

#### Statistical methods

Statistical methods employed in this study include Overall accuracy (OA), Precision, Recall, F-measure, and Matthews correlation coefficient (MCC). As shown in formula (4)–(8)^43^.

4 $$OA = \frac{TP + TN}{{TP + FP + TN + TP}}$$

5 $$Precision = \frac{TP}{{TP + FP}}$$

6 $$Recall = \frac{TP}{{TP + FN}}$$

7 $$F - measure = \frac{2 \times Precision \times Recall}{{Precision + Recall}}$$

8 $$MCC = \frac{TP \times TN - FP \times FN}{{\sqrt {(TP + FP)(TP + FN)(TN + FP)(TN + FN)} }}$$

where OA measures the ratio of correct predictions to total predictions. Precision and Recall values range between 0 and 1, with values closer to 1 indicating a higher proportion of correct predictions. In cases where Precision and Recall exhibit conflicting behavior, a commonly used approach is to consider their harmonized measure, the F-measure. MCC is essentially the correlation coefficient between actual and predicted binary classifications, yielding values ranging from −1 to + 1, where + 1 means perfect prediction, 0 means no better than random, and −1 means complete inconsistency between prediction and actual^44^.

## Landslide susceptibility mapping

### Create a training set and a validation set

**Figure 8:**
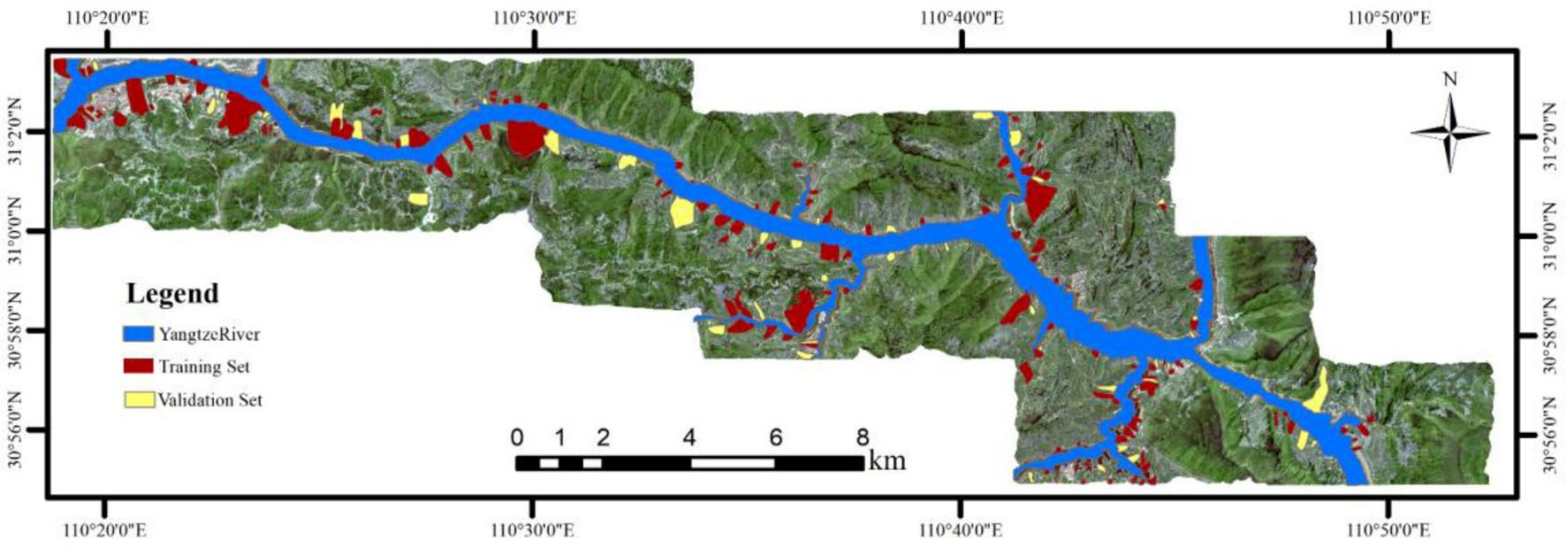
Spatial distribution of training set and validation set.

The training set was composed of an equal proportion of landslide samples (strain value of 1) and non-landslide samples (strain value of 0)^45^, Furthermore, several scholars have investigated the impact of different sample ratios in the training dataset on the outcomes of LSM^46^. Considering various LSM models, this article opted to construct the training set using an equal proportion of landslide and non-landslide samples. Taking the spatial resolution of 10 m as an example, the study area contained 3,829,404 effective grid cells, with 202 landslide occurrences. To construct the training sample set, 70% of the landslide surface were randomly selected, resulting in 141 landslides (164,274 grid cells), along with an equal number of non-landslide data (164,274 grid cells). Consequently, the training set comprised a total of 328,548 grid cells. The remaining 30% of the landslide surface (61 landslides, 68,542 grid cells) were set aside for validation. As shown in Fig. 8.

### LSM results

#### Landslide susceptibility index (LSI)

**Figure 9:**
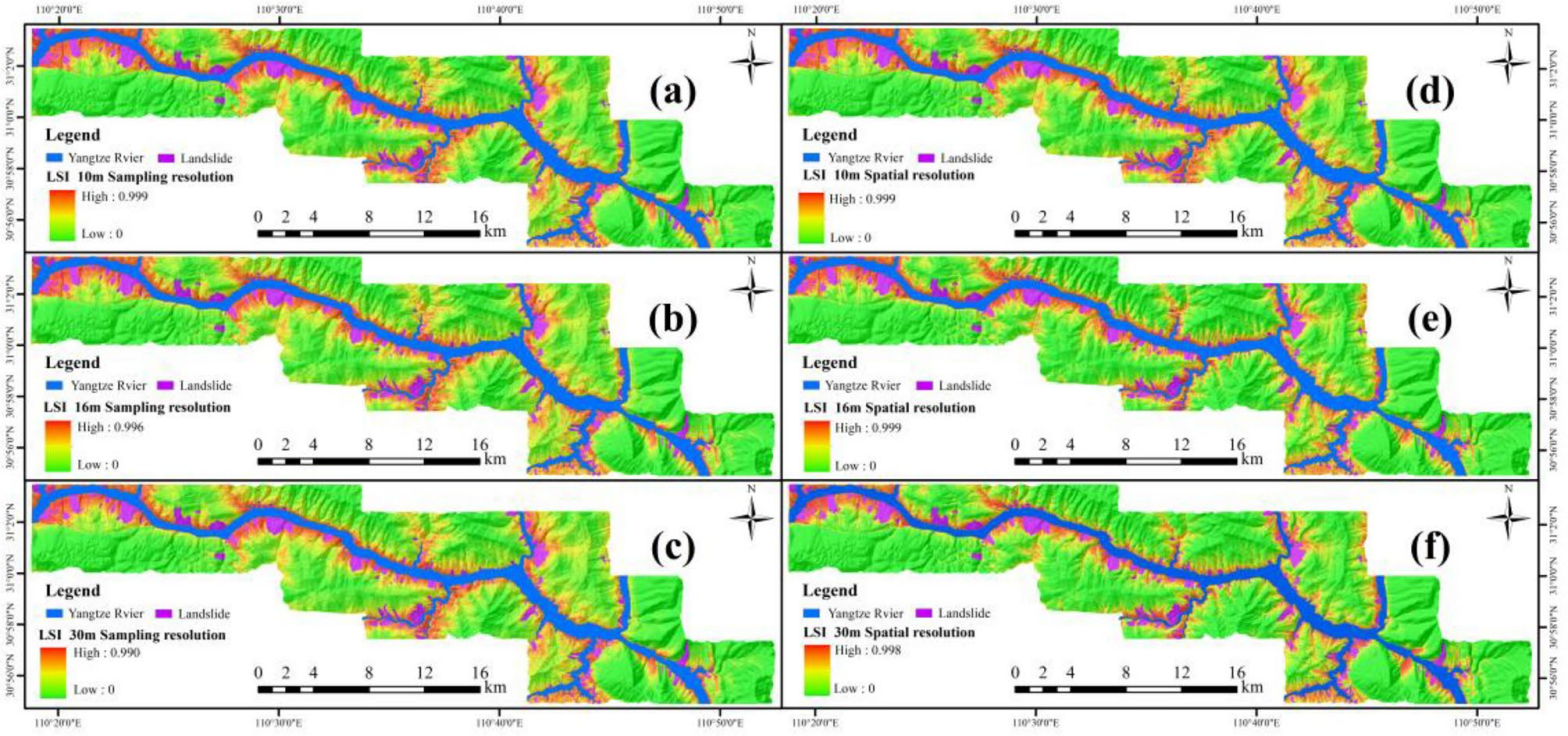
LSI produced by: (**a**) SA-10, (**b**) SA-16, (**c**) SA-30, (**d**) SP-10, (**e**) SP-16, (**f**) SP-30.

The training set constructed in "Create a training set and a validation set" was used for training the SVM model. Then, the entire set is utilized as input for the trained SVM model to generate the landslide susceptibility index (LSI) for the study area, as shown in Fig. 9.

#### Landslide susceptibility zoning (LSZ)

**Figure 10:**
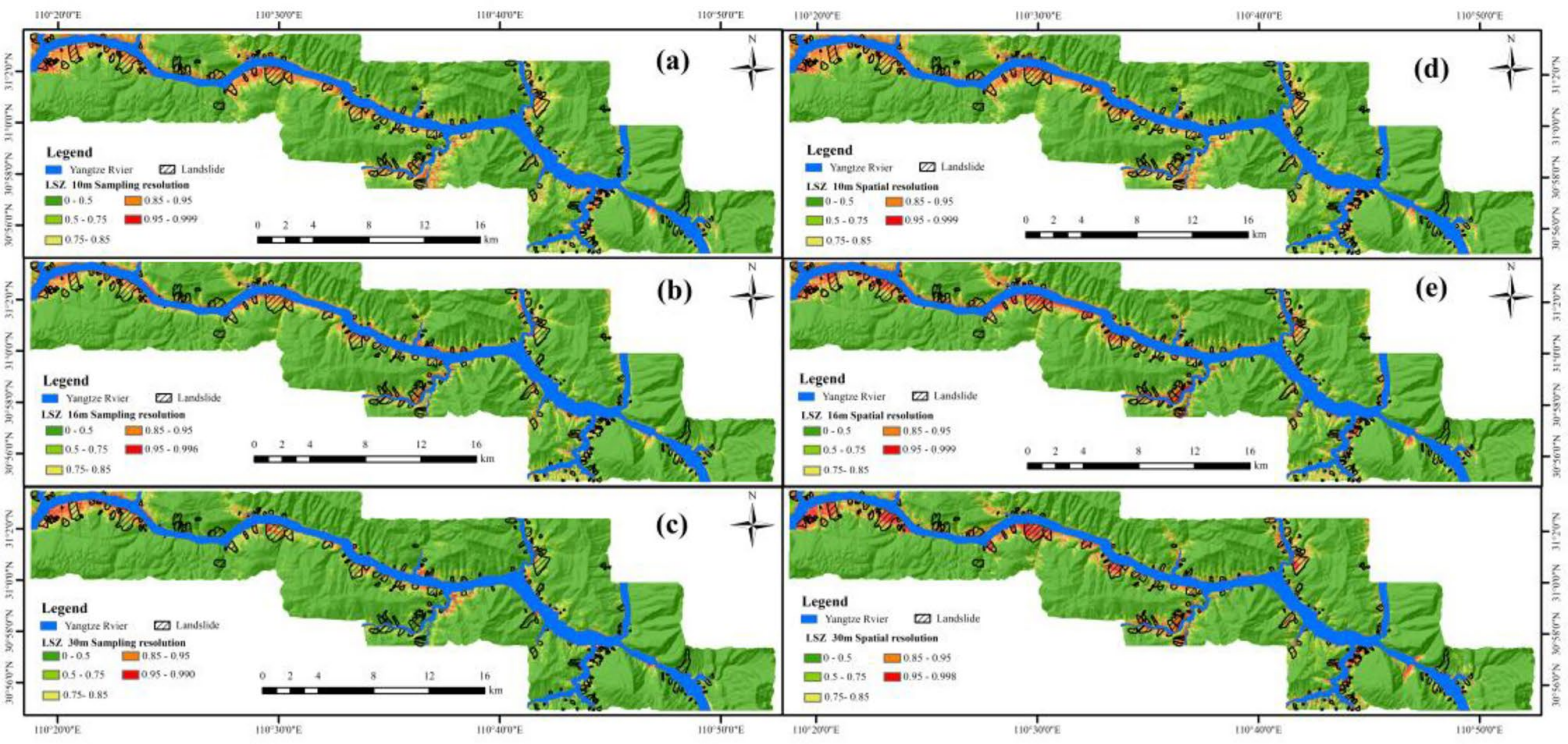
LSZ produced by: (**a**) SA-10, (**b**) SA-16, (**c**) SA-30, (**d**) SP-10, (**e**) SP-16, (**f**) SP-30.

To enhance the comprehensibility of the LSM results and provide a more intuitive representation, a manual threshold method was employed to categorize the landslide susceptibility index (LSI) map shown in Fig. 9 into five distinct levels: Very Low (0–0.5), Low (0.5–0.75), Moderate (0.75–0.85), High (0.85–0.95), and Very High (0.95–1). This classification aimed to obtained LSZ with different sampling and spatial resolutions, as shown in Fig. 10.

### Experimental results

#### ROC curve and AUC value

**Figure 11:**
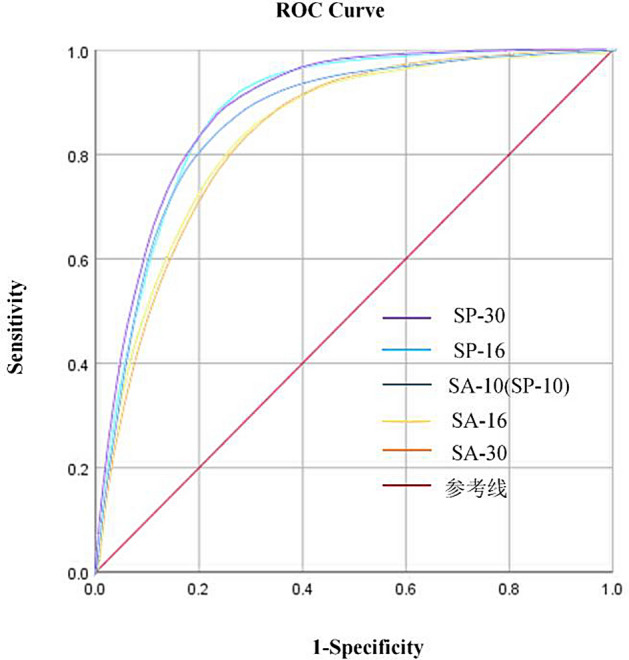
ROC curve.

The AUC corresponds to the region enclosed by the ROC curve and the X-axis. A larger AUC suggests higher precision^47^. Figure 11 and Table 5 show the ROC curves and AUC values from the SVM model at different sampling and spatial resolutions.

**Table 5:** AUC values of different sampling and spatial resolutions.

Resolution type	AUC	95% confidence interval	
		Lower limit	Upper limit
SA-10	**0.871**	0.870	0.872
SA-16	0.868	0.867	0.869
SA-30	0.866	0.864	0.868
SP-10	0.871	0.870	0.872
SP-16	0.901	0.900	0.902
SP-30	**0.913**	0.912	0.915

Maximum value is in bold.

According to Table 5, it is evident that among the sampling resolutions, the maximum AUC value of 0.871 is obtained for SA-10. Conversely, among the spatial resolutions, the highest AUC value of 0.913 is obtained for SP-30. Additionally, AUC values for spatial resolutions consistently higher than those for sampling resolutions.

#### Analysis results of specific category precision

Specific category precision based on SVM model’s experimental results at different sampling and spatial resolutions are shown in Table 6.

**Table 6:** Analysis results of specific category precision.

Resolution type	Category of susceptibility (%)				
	Very low	Low	Medium	High	Very high
SA-10	1.42	10.64	22.83	30.73	**39.14**
SA-16	1.44	11.45	22.94	29.60	38.69
SA-30	1.46	11.60	22.28	29.11	38.23
SP-10	1.42	10.64	22.83	30.73	39.14
SP-16	1.08	12.53	22.98	32.39	48.34
SP-30	1.05	12.47	22.87	33.31	**52.51**

Maximum value is in bold.

According to Table 6, the highest specific category precision in the “Very High” category for sampling resolutions was 39.14% for SA-10, while for spatial resolutions, it was 52.51% for SP-30. Additionally, spatial resolutions yielded consistently higher specific category precision in the “Very High” category than sampling resolutions.

#### Results of statistical methods

The calculation results for five statistical methods, including OA, Precision, Recall, F-measure, and MCC, are presented in Table 7.

**Table 7:** Results of calculation with statistical methods.

Resolution type	Statistical methods				
	OA (%)	Precision	Recall	F-measure	MCC
SA-10	**78.68**	**0.1985**	**0.8257**	**0.3201**	**0.3767**
SA-16	78.49	0.1979	0.8223	0.3190	0.3758
SA-30	78.28	0.1933	0.8207	0.3129	0.3708
SP-10	78.68	0.1985	0.8257	0.3201	0.3767
SP-16	81.37	0.2274	0.8633	0.3600	0.4113
SP-30	**83.00**	**0.2435**	**0.8636**	**0.3798**	**0.4286**

Maximum value is in bold.

According to Table 7, among the sampling resolutions, the 10 m resolution exhibits the highest values for OA (78.68%), Precision (0.1985), Recall (0.8257), F-measure (0.3201), and MCC (0.3767). Conversely, among the spatial resolutions, the 30 m resolution exhibits the highest values for OA (83.00%), Precision (0.2435), Recall (0.8636), F-measure (0.3798), and MCC (0.4286). Additionally, the statistical method values for spatial resolution consistently surpass those for the sampling resolution.

#### Analysis of results based on SVM for two resolutions

The results from the SVM model in Fig. 11 and Tables 5, 6 and 7 in "Experimental results" reveal that as the sampling resolution increases, the AUC value gradually decreases, the specific category precision for the “Very High” category decreases, and the performance metrics of the statistical methods (OA, Precision, Recall, F-measure, and MCC) also decline. Conversely, as the spatial resolution increases, the AUC value gradually increases, the specific category precision for the “Very High” category improves, and the performance metrics of the statistical methods also enhance. Furthermore, the AUC value, the specific category precision for the “Very High” category, and the performance metrics of the statistical methods are consistently favor spatial resolution over sampling resolution.

### Reliability analysis of the conclusions

To ensure the reliability of the impact of different sampling and spatial resolutions on LSM results, both ANN and CNN models were employed for LSM analysis. These models were trained and validated using identical datasets to ensure that any variations in the LSM results were solely attributed to changes in the models. Furthermore, AUC value, specific category precision for the “Very High” category, and statistical methods were used to analyze the experimental results. The results, as shown in Tables 8, 9 and 10 in "Analysis of AUC values for two models", “Analysis of specific category precision for two models” and "Statistical methods analysis of ANN and CNN models".

**Table 8:** AUC values for two models.

Resolution type	AUC	
	ANN	CNN
SA-10	**0.880**	**0.857**
SA-16	0.872	0.851
SA-30	0.857	0.846
SP-10	0.880	0.857
SP-16	0.911	0.885
SP-30	**0.921**	**0.890**

Maximum value is in bold.

**Table 9:** Analysis results of specific category precision for two models.

Resolution type	Specific category precision-"very high"	
	ANN	CNN
SA-10	**49.12**	**44.19**
SA-16	47.95	42.01
SA-30	45.90	41.59
SP-10	49.12	44.19
SP-16	56.49	45.18
SP-30	**56.88**	**46.41**

Maximum value is in bold.

**Table 10:** Statistical methods under ANN and CNN models.

Model	Resolution type	Statistical methods				
		OA (%)	Precision	Recall	F-measure	MCC
ANN	SA-10	**77.93**	**0.1961**	0.8488	**0.3186**	**0.3754**
	SA-16	76.51	0.1860	**0.8504**	0.3052	0.3636
	SA-30	73.79	0.1684	0.8490	0.2811	0.3422
	SP-10	77.93	0.1961	0.8488	0.3186	0.3754
	SP-16	82.05	0.2350	**0.8678**	0.3698	0.4200
	SP-30	**83.80**	**0.2534**	0.8667	**0.3921**	**0.4392**
CNN	SA-10	**80.33**	**0.2184**	**0.8044**	**0.3101**	0.3531
	SA-16	80.17	0.1998	0.7998	0.3008	**0.3662**
	SA-30	79.03	0.1895	0.7059	0.2989	0.3572
	SP-10	80.33	0.2184	0.8044	0.3101	0.3531
	SP-16	80.35	**0.2226**	0.8095	**0.3482**	**0.3998**
	SP-30	**80.68**	0.2071	**0.8382**	0.3321	0.3873

Maximum value is in bold.

#### Analysis of AUC values for two models

According to Table 8, it is evident that both the ANN and CNN models exhibit results that closely resemble those of the SVM model. Among the sampling resolutions, the highest AUC values were achieved at a 10 m resolution, with respective values of 0.880 and 0.857. Moreover, the AUC values obtained by the models gradually decrease as the sampling resolution increases. Concerning spatial resolution, the highest AUC values were achieved at a 30 m resolution, with respective values of 0.921 and 0.890. Furthermore, the AUC values obtained by the models gradually increase as the spatial resolution increases. Additionally, AUC values for spatial resolution consistently surpass those for sampling resolution.

#### Analysis of specific category precision for two models

According to Table 9, it is evident that both the ANN and CNN models exhibit results that closely resemble those of the SVM model. Among the sampling resolution, the specific category precision for the “Very High” category was achieved at a 10 m resolution, with respective values of values of 49.12% and 44.19%. Moreover, the specific category precision obtained by the models gradually decrease as the sampling resolution increases. Regarding spatial resolution, the specific category precision for the “Very High” category were achieved at a 30 m resolution, with respective values of 56.88% and 46.41%. Furthermore, the specific category precision obtained by the models gradually increase as the spatial resolution increases. Additionally, the specific category precision for the “Very High” category for spatial resolution are surpasses that in sampling resolution.

#### Statistical methods analysis of ANN and CNN models

According to Table 10, it is evident that both the ANN and CNN models exhibit results that are slightly differ from those of the SVM model. In the validation results of the ANN model, the highest OA (77.93%), Precision (0.1961), F-measure (0.3186), and MCC (0.3754) were achieved at a resolution of 10 m for sampling resolution, with only the Recall (0.8504) being highest at a resolution of 16 m. For spatial resolution, the highest OA (83.80%), Precision (0.2534), F-measure (0.3921), and MCC (0.4392) were achieved at a resolution of 30 m, with only the Recall (0.8678) being highest at a resolution of 16 m. In the validation results of the CNN model, the highest OA (80.33%), Precision (0.2184), Recall (0.8044), and F-measure (0.3101) were achieved at a resolution of 10 m for sampling resolution, only the MCC (0.3662) was highest at a resolution of 16 m. For spatial resolution, the highest OA (80.68%) and Recall (0.8382) were achieved at a resolution of 30 m, the highest Precision (0.2226), F-measure (0.3482), and MCC (0.3998) were achieved at a resolution of 16 m. Additionally, the OA, Precision, Recall, F-measure, and MCC values for spatial resolution exceed those for sampling resolution.

#### Quantitative analysis of LSM results

Given the inconsistent results among the SVM, ANN, and CNN models using statistical methods, a scoring method was employed to provide a comprehensive evaluation of the impact of different sampling and spatial resolutions on LSM. This method involved evaluating the AUC value of LSM results, the specific category precision for the “Very High” category, and statistical methods. The scoring principle is as follows: the AUC value, the specific category precision for the “Very High” category, and statistical methods obtained from experiments with different sampling and spatial resolutions are ranked from high to low and assigned scores ranging from 3 to 1, with 3 being the highest score and 1 the lowest. In the case of ties, the lower score is assigned. For statistical methods, the score is determined by averaging the scores obtained from the five methods: OA, Precision, Recall, F-measure, and MCC. A higher score in the quantitative analysis indicates a higher level of prediction accuracy^24^.The score tables are shown in Tables 11 and 12.

**Table 11:** Comprehensive quantitative evaluation results for sampling resolution.

Model	Sampling resolution	AUC	Specific category precision “very high”	Statistical methods	Total score
SVM	10	3	3	3	**9**
	16	2	2	2	6
	30	1	1	1	3
ANN	10	3	3	2.6	**8.6**
	16	2	2	2.2	6.2
	30	1	1	1.2	3.2
CNN	10	3	3	2.6	**8.6**
	16	2	2	2.2	6.2
	30	1	1	1.2	3.2

Maximum value is in bold.

**Table 12:** Comprehensive quantitative evaluation results for spatial resolution.

Model	Spatial resolution	AUC	Specific category precision “very high”	Statistical methods	Total score
SVM	10	1	1	1	3
	16	2	2	2	6
	30	3	3	3	**9**
ANN	10	1	1	1	3
	16	2	2	2.2	6.2
	30	3	3	2.8	**8.8**
CNN	10	1	1	1.2	4.2
	16	2	2	2.8	6.8
	30	3	3	2.2	**8.2**

Maximum value is in bold.

According to Tables 11 and 12, it can be observed that for sampling resolution, the highest integrated scores of AUC value, specific category precision for the "Very High" category, and the average value of statistical methods in the comprehensive quantitative evaluation results of the SVM, ANN, and CNN models were obtained at a sampling resolution of 10 m, with scores of 9, 8.6, and 8.6, respectively. Regarding spatial resolution, the highest integrated scores of AUC value, specific category precision for the “Very High” category, and the average value of statistical methods in the comprehensive quantitative evaluation results of the SVM, ANN, and CNN models were obtained at a spatial resolution of 30 m, with scores of 9, 8.8, and 8.2, respectively.

Based on comprehensive quantitative analysis, it has been observed that the prediction accuracy of LSM results decreases with an increase in sampling resolution, while it increases with an increase in spatial resolution. Moreover, the values of AUC, specific category precision for the “Very High” category, and statistical methods at the three different spatial resolutions are higher than those at the sampling resolution, indicating that the impact of spatial resolution on LSM results is greater than that of sampling resolution.

### Further experiments on sampling resolution

**Figure 12:**
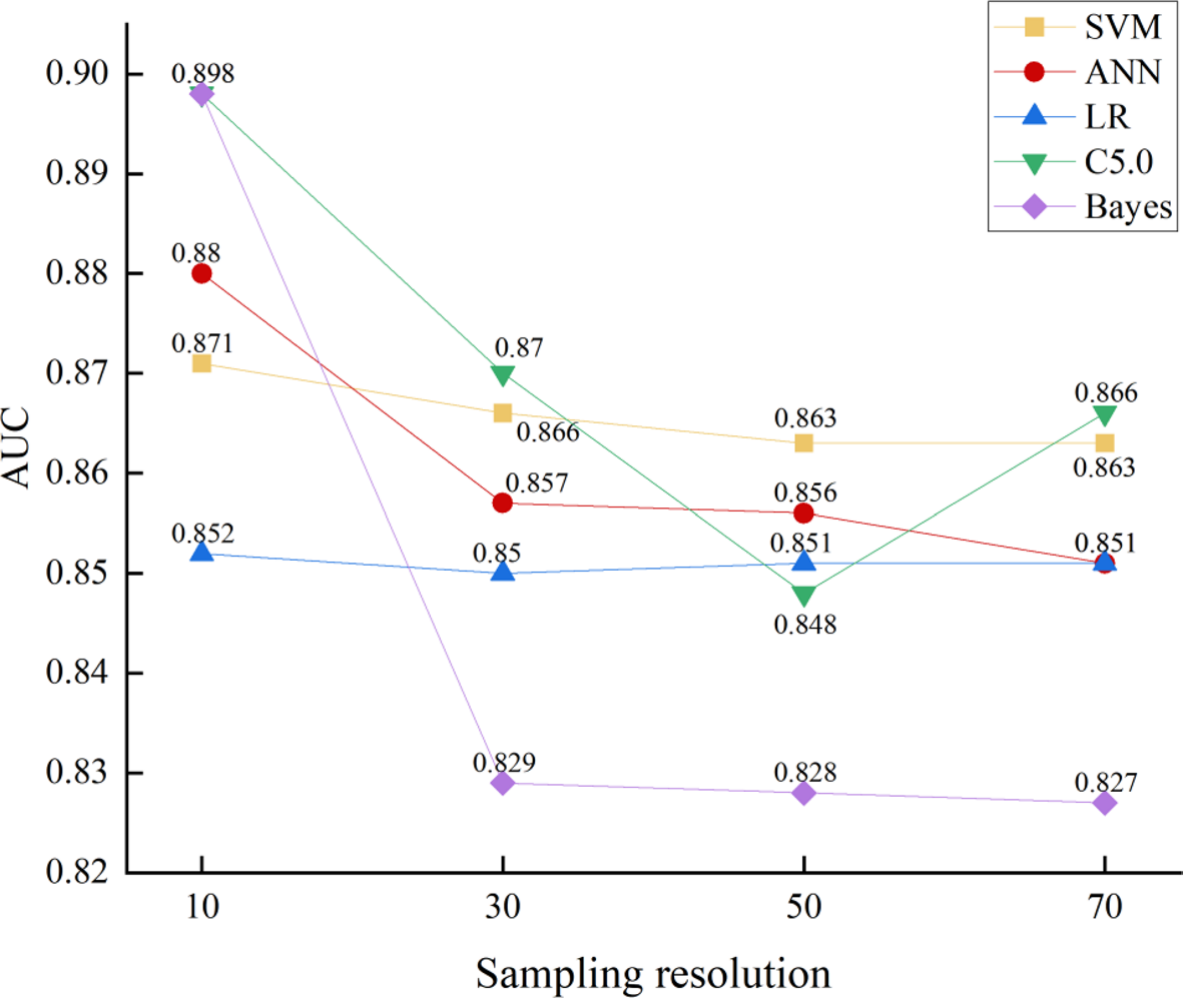
AUC values.

**Figure 13:**
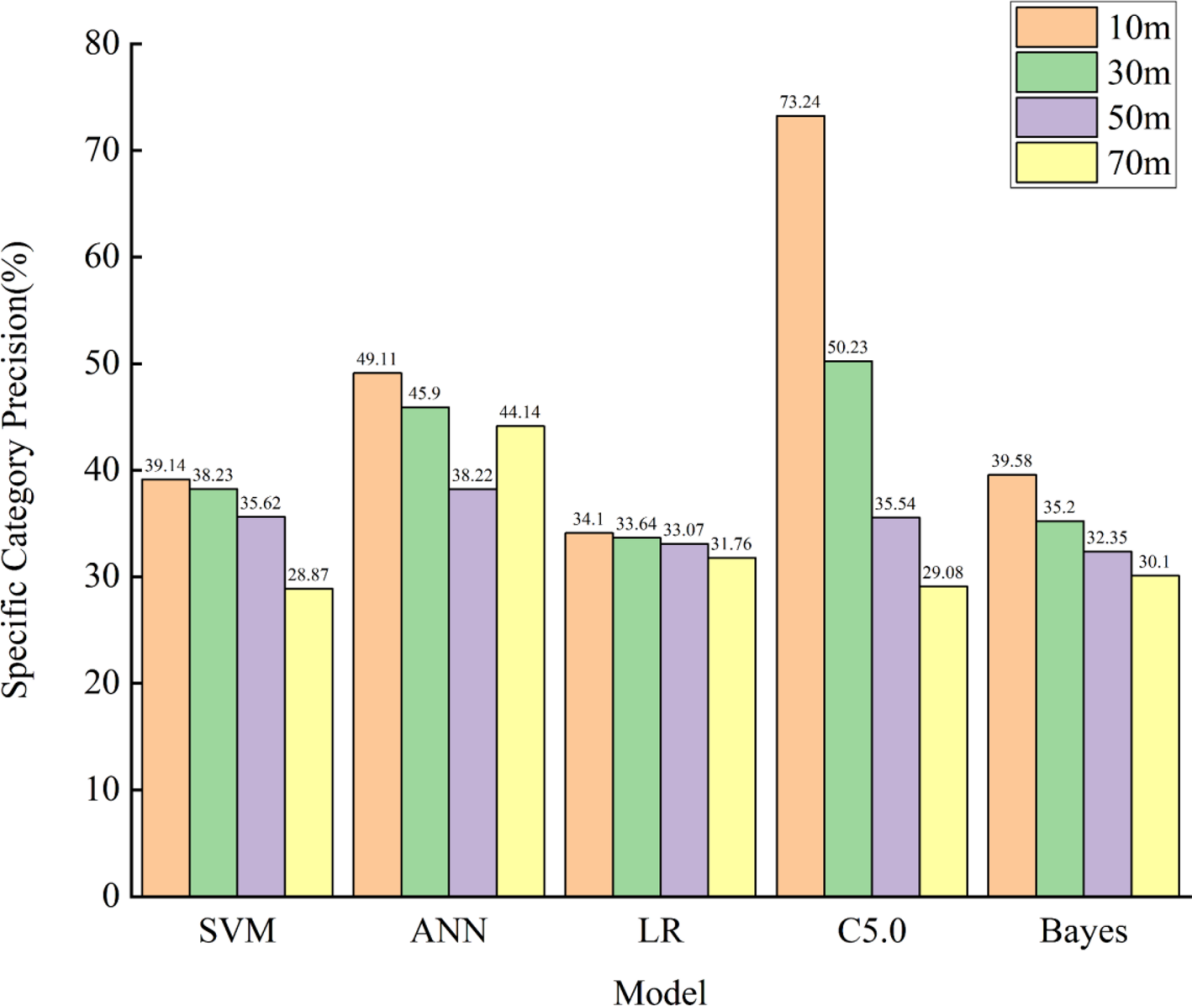
Specific category precision “Very High”.

Given the absence of relevant research on the effect of sampling resolution on LSM results, this study will further investigate the performance results of different sampling resolutions under various machine learning models to verify the impact of sampling resolution on LSM results. This study includes five widely employed machine learning models: SVM, ANN, LR, C5.0, and Bayes, to model different sampling resolutions (10 m, 30 m, 50 m, and 70 m). The LSM results will be evaluated using AUC values, specific category precision, and statistical methods. Furthermore, a comprehensive quantitative assessment will be conducted through the utilization of a scoring method. The results of AUC values and specific category precision are shown in Figs. 12 and 13, while Tables 13 and 14 provide the statistical methods and score tables.

**Table 13:** Statistical methods under five models.

Model	Resolution	Statistical methods				
		OA (%)	Precision	Recall	F-measure	MCC
SVM	10	**78.68**	**0.1985**	**0.8257**	**0.3201**	**0.3767**
	30	78.28	0.1933	0.8207	0.3129	0.3708
	50	78.10	0.1910	0.8165	0.3095	0.3679
	70	77.33	0.1869	0.8255	0.3048	0.3638
ANN	10	**77.93**	**0.1961**	0.8487	**0.3186**	**0.3754**
	30	73.79	0.1684	**0.8499**	0.2821	0.3422
	50	74.75	0.1701	0.8244	0.2819	0.3439
	70	73.72	0.1664	0.8238	0.2725	0.3580
LR	10	**76.57**	**0.1789**	**0.7948**	**0.2920**	0.3530
	30	76.17	0.1766	0.7924	0.2861	**0.3581**
	50	76.12	0.1776	0.7908	0.2900	0.3514
	70	76.13	0.1762	0.7907	0.2886	0.3501
C5.0	10	**96.38**	**0.6816**	0.7317	**0.7111**	**0.7032**
	30	91.94	0.4114	0.7826	0.5393	0.5543
	50	87.15	0.2927	0.8031	0.4290	0.4661
	70	84.39	0.2567	**0.8406**	0.3933	0.4391
Bayes	10	74.57	0.1623	0.7648	0.2678	0.3327
	30	**75.13**	**0.1650**	0.7700	**0.2718**	**0.3362**
	50	73.95	0.1601	0.7845	0.2657	0.3310
	70	73.65	0.1590	**0.7870**	0.2465	0.3296

Maximum value is in bold.

**Table 14:** Comprehensive quantitative evaluation results.

Model	Resolution	AUC	Specific category precision “very high”	Statistical methods	Total score
SVM	10	4	4	4	**12**
	30	3	3	3	9
	50	2	2	1.8	5.8
	70	1	1	1.2	3.2
ANN	10	4	4	3.8	**11.8**
	30	3	3	2.4	8.4
	50	2	1	2.4	5.4
	70	1	2	1.4	4.4
LR	10	4	4	3.8	**11.8**
	30	1	3	2.6	6.6
	50	2	2	2.4	6.4
	70	2	1	1.2	4.2
C5.0	10	4	4	3.4	**11.4**
	30	3	3	2.8	8.8
	50	1	2	2.2	5.2
	70	2	1	1.6	4.6
Bayes	10	4	4	2.6	**10.6**
	30	3	2	3.6	8.6
	50	1	3	2.2	6.2
	70	2	1	1.6	4.6

Maximum value is in bold.

In response to the results of Figs. 12 and 13 and Table 13, a comprehensive quantitative assessment will be made using a scoring method, as shown in Table 14.

According to Table 14, the comprehensive quantitative evaluation results of SVM, ANN, LR, C5.0, and Bayes models indicate that, overall, AUC value, specific category precision for the “Very High” category, and statistical methods average, the highest comprehensive score of the three is 10 m sampling resolution, which are 12, 11.8, 11.8, 11.8, and 10.6, respectively, Moreover, the total score decreases as the sampling resolution increases, Based on the above results, it is demonstrated that the prediction accuracy of the LSM results decreases as the sampling resolution increases, consistent with the findings in "Reliability analysis of the conclusions"; thus, validating the effect of sampling resolution on the results of landslide susceptibility assessment.

## Discussion

**Figure 14:**
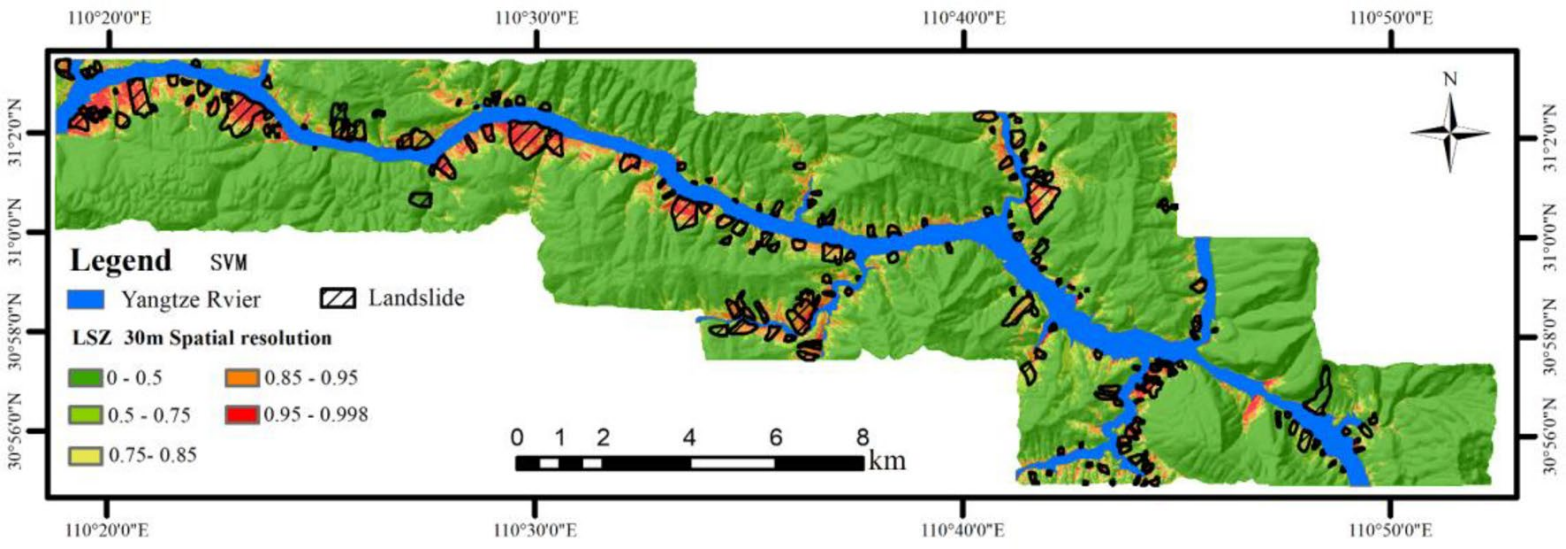
LSZ chart for SA-30.

The LSZ map obtained from the SVM model at 30 m spatial resolution is chosen as an example for analysis. It is shown in Fig. 14.

As can be seen from Fig. 14, there are 21 km^2^ of high susceptibility areas, accounting for 5.41% of the total area, and 12 km^2^ of very high susceptibility areas, accounting for 3.09% of the total area. In addition, the medium, high, and very high susceptibility subzones are mainly located along the river, and the low and very low susceptibility subzones are mainly located further away from the water system. Through field surveys, it is evident that landslides are primarily located in the medium, high, and very high susceptibility zones, accounting for 95.05% of the total. Similar conclusions were found in the LSM results at other resolutions, indicating that the experimental results obtained are consistent with the distribution of landslides and engineering experience in the study area.

**Figure 15:**
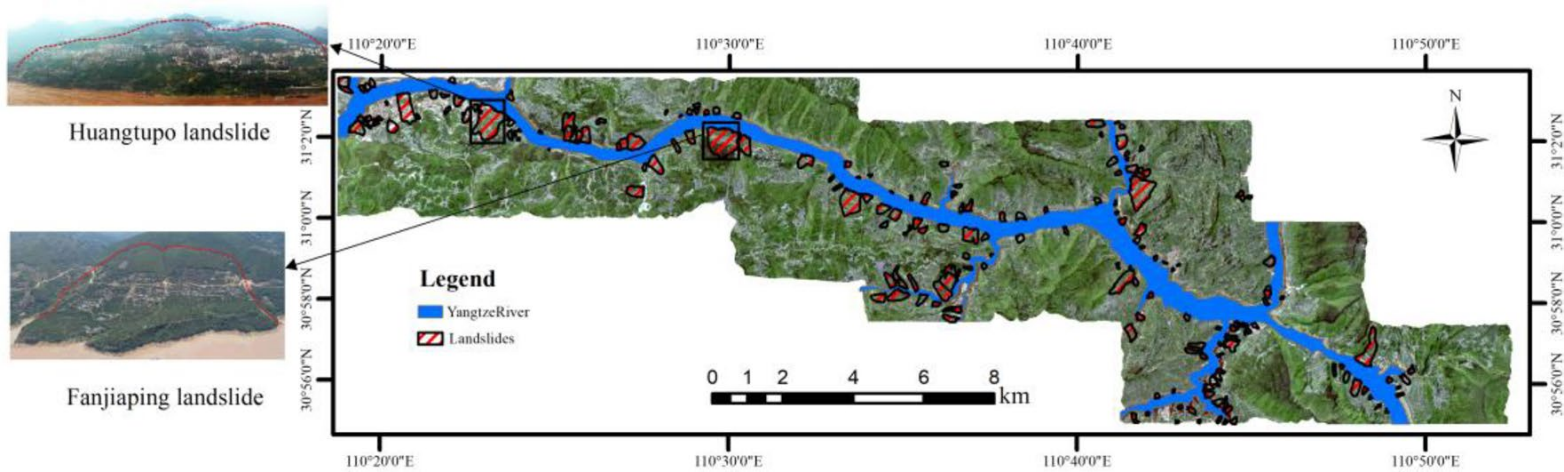
Distribution of landslides. (The pictures of the Huangtupo landslide and the Fanjiaping landslide on the left are taken at the scene, Remote sensing imagery from publicly available Sentinel-2 satellite imagery; https://sentinel.esa.int/).

In order to further validate the conclusions obtained in this article, Fanjiaping landslide and Huangtupo landslide were selected for comparative analysis and validation of the results. Their distribution is shown in Fig. 15.

**Figure 16:**
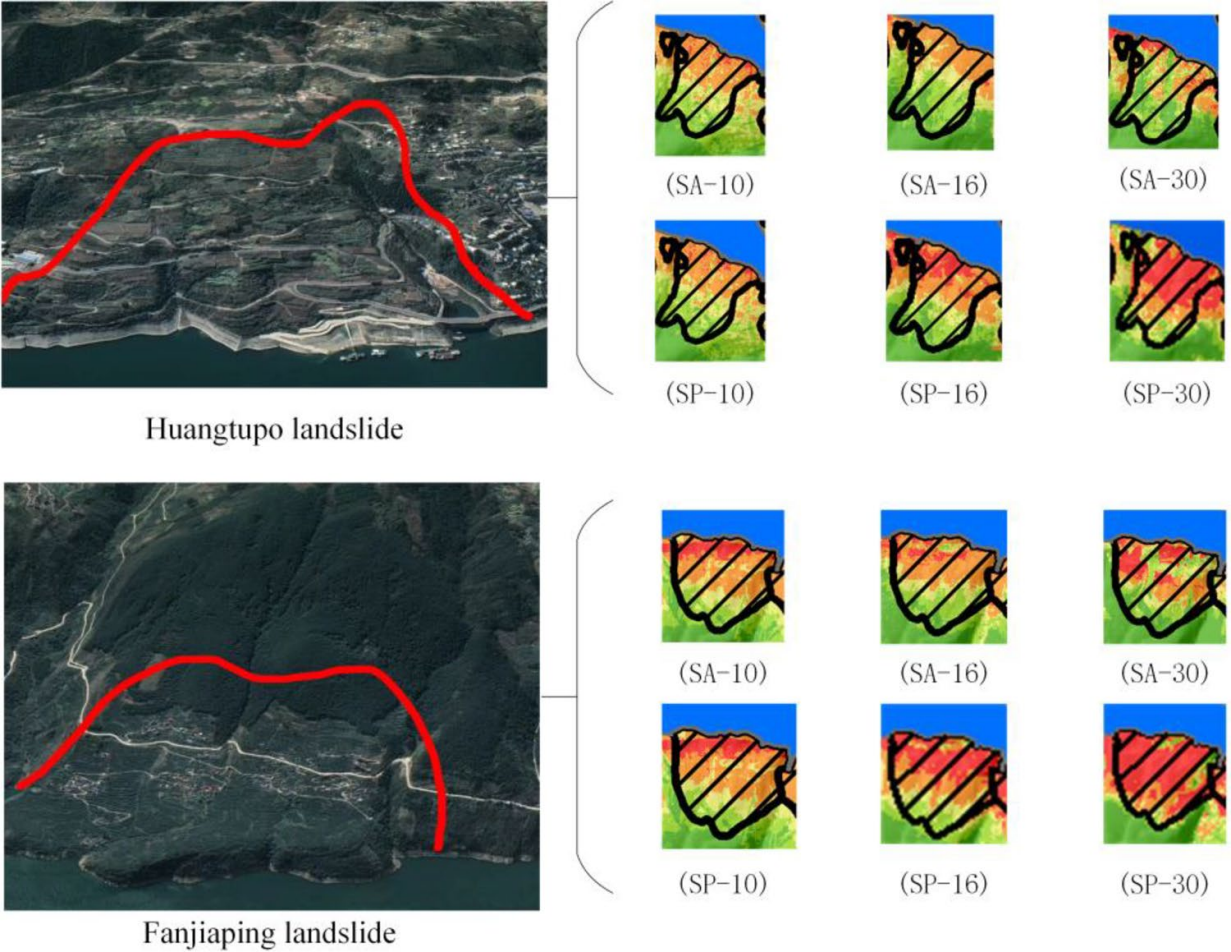
Detailed map of the Huangtupo landslide and Fanjiaping landslide (satellite images from Google Earth Pro 7.3; https://www.google.com/intl/en/earth/).

Combining the distribution of known landslide surfaces in the study area (Fig. 15) and the results of LSZ (Fig. 10), and selecting the Huangtuopo landslide and the Fanjiaping landslide as a reference to get Fig. 16.

In the case of the Huangtupo landslide, the northwest corner of SA-30 exhibits the highest prediction accuracy, and the degree of agreement of its prediction results gradually increases as the sampling resolution decreases. Regarding SP-30, it displays the highest agreement in prediction results, while the north and northwest corners of SP-16 have higher prediction accuracies, with the agreement of their prediction results gradually decreasing as the spatial resolution decreases. For the Fanjiaping landslide, higher prediction accuracies are observed in the northern, central, and northwestern corners of SA-30, with the degree of agreement of its prediction results increasing as the sampling resolution decreases. In the case of SP-30, it includes almost the entire landslide surface, and the degree of agreement gradually decreases with the decrease in spatial resolution. Moreover, the predictions at spatial resolutions tend to match more closely than those at their corresponding sampling resolutions.

The analysis of the experimental results in "Experimental results" and "Reliability analysis of the conclusions" has led to the conclusion that the highest prediction accuracy is achieved with a 10 m sampling resolution. Since there is no relevant study on the effect of sampling resolution on LSM results to corroborate these findings, this study further explores the impact of sampling resolution in "Further experiments on sampling resolution", which yields experimental results that align with the previous conclusions. This further validates the influence that sampling resolution has on LSM results. This influence may be attributed to the influence of the number of training sample points with 10 m sampling resolution is sufficiently large compared to the sampling resolutions of 16 m, 30 m, 50 m and 70 m, and a sufficient number of training sample points can construct a model that is more relevant to the actual situation. However, it is important to note that the difference in sampling resolution also affects the spatial correlation between sample points. When conducting statistical analyses of data with spatial attributes, it is necessary to consider the interdependence of training samples, and the spatial correlation between the samples should be reduced as much as possible^48^. Therefore, in future studies, it is important to explore a suitable sampling resolution that can reduce the spatial correlation between sample points while ensuring a sufficient number of training sample points for the analysis of LSM results.

The analysis of experimental results in "Experimental results" and "Reliability analysis of the conclusions" indicates that a 30 m spatial resolution yields the highest prediction accuracy. This finding diverges from several existing studies, possibly due to the influence of spatial resolution on landslides being contingent on the scale of landslides within the study area. Utilizing identical resolutions for both landslides and geo-environmental information may introduce potential biases^49^. For example, small grid cells adeptly capture morphological details of shallow landslides but are less effective for large, deep-seated landslides, whose features are more discernible at coarser resolutions^50,51^. The study also acknowledges certain limitations: (1) Landslides, as complex natural hazards, are influenced by various geological and environmental factors, complicating accurate modeling; (2) The quality of the DEM data; (3) The limited number of landslide references in the database. Consequently, further research on the optimal spatial resolution for predictive LSM modeling is warranted.

## Conclusion

This article focuses on the section from Zigui to Badong in the Three Gorges Reservoir Area as the study area. The SVM model is employed to generate LSM results under various sampling and spatial resolutions. The obtained results are then evaluated and analyzed using ROC curves, specific category accuracy, and statistical methods. To ensure the reliability of the experimental findings, ANN and CNN models were also used for verification. Subsequently, a comprehensive quantitative scoring method is employed to assess the LSM results from obtained the three models. To verify the reliability of the sampling resolution results, five models of SVM, ANN, LR, C5.0 and Bayes were selected to model and discuss four different sampling resolutions (10 m, 30 m, 50 m and 70 m). The total score results indicate that the highest sampling resolution of 10 m yields the best prediction accuracy for LSM results. As the sampling resolution increases, the prediction accuracy of LSM results decreases, consistent with the experimental results obtained in "Reliability analysis of the conclusions". The results show that: firstly, the results of 10 m sampling resolution in SVM, ANN, and CNN models outperform those at 16 m and 30 m. As the sampling resolution increases, the accuracy of LSM result predictions decreases. Conversely, the results of 30 m spatial resolution in SVM, ANN, and CNN models are superior to those at 10 m and 16 m. Moreover, as the spatial resolution increases, the accuracy of LSM result predictions increases. Secondly, AUC values, specific category precision for the “Very High” category, and statistical methods results derived from the spatial resolution are superior to those obtained from the sampling resolution. This indicates that spatial resolution has a greater impact on the LSM results than sampling resolution. Finally, Fanjiaping landslide and Huangtupo landslide are selected as references for comparative analysis and verification of the results, and the results obtained are in line with the engineering reality.

This article provides systematic research on different sampling and spatial resolutions, which can provide a certain degree of reference for the selection of sampling resolution and spatial resolution of LSM factors when researchers carry out LSM. These findings contribute to improving the scientific accuracy and precision of LSM, holding significant theoretical and practical value for engineering applications.

## Data Availability

The data processing platform can be downloaded directly through the link provided in Table 3. However, basic geographic data, basic geological data, and landslide distribution data are all confidential data in China. According to the requirements of relevant laws, these confidential data have been decrypted when we use them. Any researchers in related fields that need these decrypted data can contact the corresponding author to obtain them.
